# Inhibition of Mild Steel Corrosion in Sulfuric Acid Solution by New Schiff Base

**DOI:** 10.3390/ma7020787

**Published:** 2014-01-28

**Authors:** Ahmed A. Al-Amiery, Abdul Amir H. Kadhum, Abdulhadi Kadihum, Abu Bakar Mohamad, Chong K. How, Sutiana Junaedi

**Affiliations:** 1 Department of Chemical & Process Engineering, University Kebangsaan Malaysia (UKM), Bangi, Selangor 43000, Malaysia; E-Mails: amir@eng.ukm.my (A.A.H.K.); drab@eng.ukm.my (A.B.M.); howardchong90@gmail.com (C.K.H.); sutianajnd10@gmail.com (S.J.); 2 Applied Science Department, University of Technology (UOT), Baghdad 10001, Iraq; E-Mail: abdulhadikadhim5@gmail.com (A.K.)

**Keywords:** electrochemical measurements, SEM, corrosion inhibitor

## Abstract

The efficiency of Schiff base derived from 4-aminoantipyrine, namely 2-(1,5-dimethyl-4-(2-methylbenzylidene)amino)-2-phenyl-1H-pyrazol-3(2H)-ylidene) hydrazinecarbothioamide as a corrosion inhibitor on mild steel in 1.0 M H_2_SO_4_ was investigated using electrochemical impedance spectroscopy (EIS), potentiodynamic polarization (PD) and electrochemical frequently modulation (EFM) in addition to the adsorption isotherm, corrosion kinetic parameters and scanning electron microscopy (SEM). The results showed that this inhibitor behaved as a good corrosion inhibitor, even at low concentration, with a mean efficiency of 93% and, also, a reduction of the inhibition efficiency as the solution temperature increases. A polarization technique and EIS were tested for different concentrations and different temperatures to reveal that this compound is adsorbed on the mild steel, therefore blocking the active sites, and the adsorption follows the Langmuir adsorption isotherm model. The excellent inhibition effectiveness of 2-(1,5-dimethyl-4-(2-methylbenzylidene)amino)-2-phenyl-1H-pyrazol-3(2H)-ylidene)hydrazinecarbothioamide was also verified by scanning electron microscope (SEM).

## 1. Introduction

Inhibitors are chemical compounds added in small quantities in order to reduce the corrosion rate [1]. The presence of such compounds retards the corrosion process and keeps its rate to a minimum and, thus, prevents economic losses due to metallic corrosion. The chemicals that can act as corrosion inhibitors may be inorganic or organic [2]. Inhibitors slow corrosion processes [3] by:

- Reducing the anodic and cathodic reaction speed;
- Reducing the movement or diffusion of ions to and from the metallic surface;
- Increasing the electrical resistance of the metal surface.

Organic corrosion inhibitors are an attractive field of research, due to their usefulness in various industries. The efficiency of an inhibitor depends on the stability of the formed chelate, and the inhibitor molecule should have centers that are capable of forming bonds with the metal surface via an electron transfer [4]. Most organic inhibitors adsorb on the metal surface by displacing water molecules on the surface and forming a compact barrier. The availability of non-bonded (lone pair) and *p*-electrons in inhibitor molecules facilitate the electron transfer from the inhibitor to the metal [5]. The efficiency of the inhibitor depends on the stability of the chelate formed [6], so it mainly depends on the type and the nature of the substituents present in the inhibitor molecule [7]. The choice of the inhibitors is based on two considerations: first, they could be synthesized conventionally from relatively cheap (our inhibitor synthesis from thiosemicarbazide) raw materials; second, they contain the electron clouds or the electronegative atoms [8,9]. A coordinate covalent bond involving the transfer of electrons from the inhibitor to the metal surface may be formed [10]. The strength of the chemisorption bond depends upon the electron density on the donor atom of the functional group and also the polarizability of the group. When an H atom attached to the C in the ring (heterocyclic ring) is replaced by a substituent group (–NH_2_, –NO, –CHO or –COOH), it improves the inhibition [11]. The electron density of the metal at the point of attachment changes, resulting in the retardation of cathodic or anodic reactions. Electrons are consumed at the cathode and are furnished at the anode [12]. Generally, a strong coordination bond causes higher inhibition efficiency, the inhibition increasing in the sequence O < N < S < P [13]. Organic inhibitors generally have heteroatoms. O, N and S are found to have higher basicity and electron density and, thus, act as corrosion inhibitors. O, N and S are the active centers for the process of adsorption on the metal surface [14]. The inhibitor molecule should have centers capable of forming bonds with the metal surface of the electron transfer, in which the metal acts as an electrophile and the inhibitor acts as a Lewis base, whose nucleophilic centers are O and/or N atoms with free electron pairs, which are readily available for sharing. Schiff base compounds are a condensation product of an amine and a ketone/aldehyde. Schiff base inhibitors have been reported as effective corrosion inhibitors for steel, copper and aluminum [15–17]. However, the presence of a hydrophilic functional group in the molecule would increase the solubility of the inhibitors [18]. The effectiveness of an organic substance as an inhibitor depends on the structure of the inhibitor [19]. The aim of the present investigation is to examine the inhibitory action of Schiff base derived from 4-aminoantipyrine and thiosemicarbazide for the corrosion of mild steel in 1.0 M H_2_SO_4_ solution. The effects of concentrations, temperatures and molecular structures on the inhibition efficiencies of the Schiff base have been studied systematically.

## 2. Results and Discussion

### 2.1. Electrochemical Impedance Spectroscopy (EIS)

**Figure 1.**
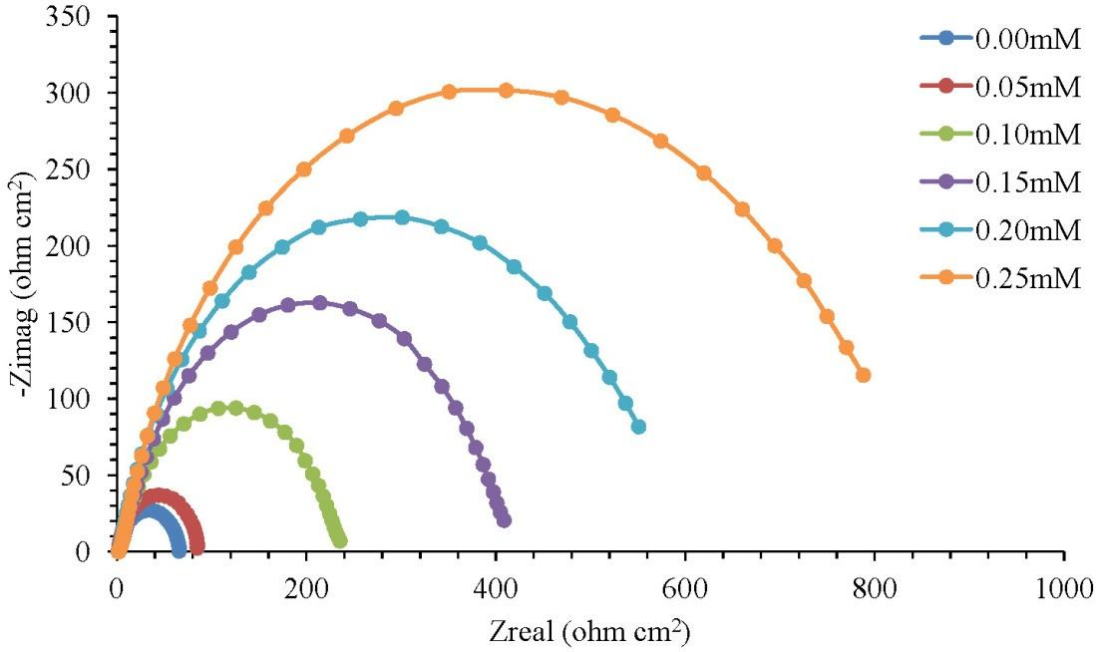
Nyquist plot for mild steel in 1.0 M H_2_SO_4_ with different concentrations of the corrosion inhibitor at 30 °C.

**Figure 2.**
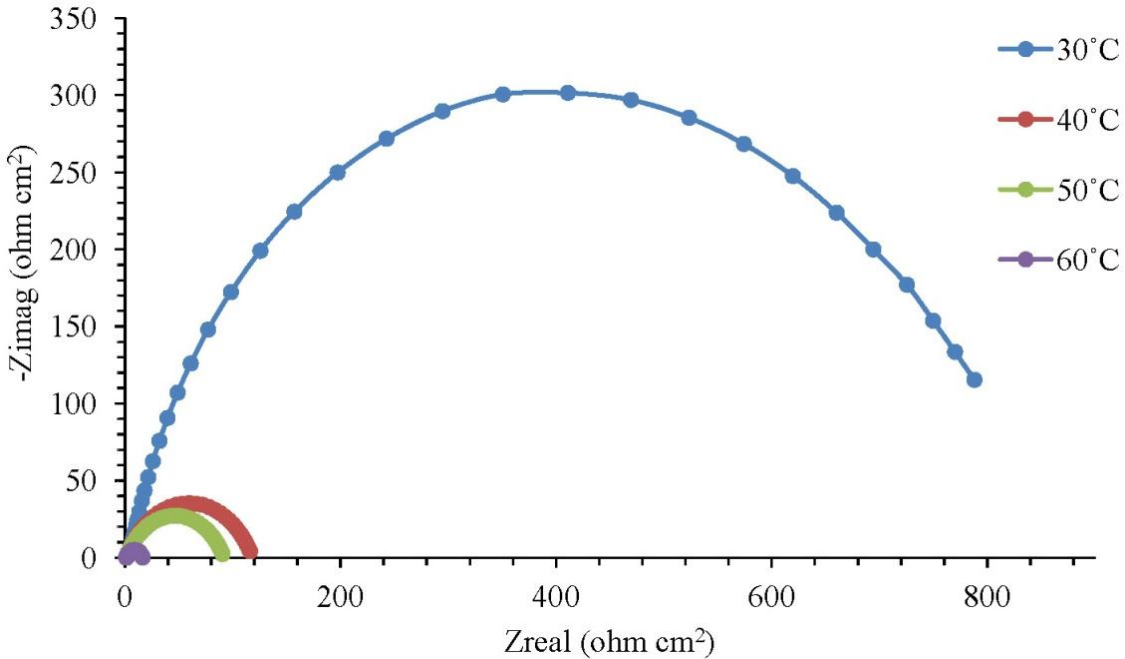
Nyquist plot for mild steel in 1.0 M H_2_SO_4_ with 0.25 mM of the corrosion inhibitor at different temperatures.

The corrosion of mild steel in 1.0 M H_2_SO_4_ solution in the presence of corrosion inhibitors was studied at temperatures of 30 °C, 40 °C, 50 °C and 60 °C. Figure 1 illustrates the Nyquist plots for mild steel in 1.0 M H_2_SO_4_ with different concentrations of the corrosion inhibitor at 30 °C, while Figure 2 represents the Nyquist plots for mild steel in 1.0 M H_2_SO_4_ containing 0.25 mM of the corrosion inhibitor at different temperatures.

Based on Figure 1, the increase in resistance can be detected significantly with increasing concentrations of the corrosion inhibitor. Each concentration of the corrosion inhibitor has a distinctive semicircular graph. A semicircle diameter can be associated with the rate of corrosion inhibition. The semicircle diameter increases with the increase in the concentration of the corrosion inhibitor, which means that the corrosion rate decreases. In other words, the rate of corrosion inhibition is increasing. Based on Figure 2, the diameter of the semicircle becomes smaller as the temperature is increased to 30 °C and to 60 °C. The higher the temperature of the solution, the smaller the diameter of the semicircle. This result means that the rate of corrosion inhibition is decreasing with the solution temperature increasing. The semicircle in Figures 1 and 2 is less perfect when the corrosion inhibitor concentration increases (in the solution), due to roughness and other imperfections on the surface of the mild steel samples and the phenomenon known as the “dispersing effect” [20]. Gamry Echem Analyst software can analyze the data of electrochemical impedance spectroscopy (EIS) experiments, such as the *CPE* matching, calculating the solution resistance, *R_s_*, the constant phase element, *CPE*, the charge transfer of resistance, *R_ct_*, and the double-layer charge, *C_dl_*. Table 1 shows the *CPE* matching data for mild steel in 1.0 M H_2_SO_4_ with different concentrations of the corrosion inhibitor at 30 °C, whereas Table 2 shows the *CPE* matching data for mild steel in 1.0 M H_2_SO_4_ having 0.25 mM of the corrosion inhibitor at different temperatures. Based on Table 1, the *R_ct_* value increases with the increasing concentration of the corrosion inhibitor. This means that the corrosion inhibitor molecules were adsorbed on the surface of mild steel samples and form a film that protects the mild steel samples. Therefore, the inhibition efficiency (*IE*) increases with the increasing concentration of the corrosion inhibitor. Based on Table 2, an increase in temperature from 30 °C to 60 °C has caused the *R_ct_* and *IE* to decrease. The corrosion inhibitor molecules adsorbed on a metal surface will experience desorption if the solution temperature is increasing.

**Scheme 1.**
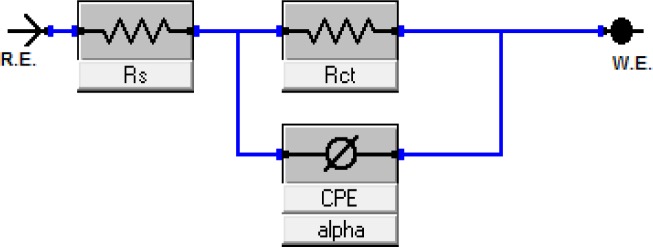
The equivalent circuit model used to fit the impedance data for mild steel in the presence of the inhibitor.

Once the results of the electrochemical impedance spectroscopy (EIS) were measured, the matching of the circuit was performed using the equivalent circuit model. A *CPE* circuit was selected as an equivalent model to do the matching. In Scheme 1, *R_s_* is the solution resistance, *R_ct_* is the charge transfer resistance and *CPE* is the constant phase element.

### 2.2. Potentiodynamic Polarization

The electrochemical kinetic parameters obtained from Tafel line extrapolation, such as corrosion current density (*i_corr_*), corrosion potential (*E_corr_*), corrosion rate and anodic and cathodic Tafel slope (βa and βc), are shown in Table 3. Inhibition efficiency (*IE*) is calculated by the following formula:

$$IE(\%)=\frac{i_{corr}^{0}-i_{corr}}{i_{corr}^{0}}\times 100$$ (1)

where *i_corr_*^0^ and *i_corr_* are the corrosion current density values without and with the corrosion inhibitor, respectively.

**Figure 3.**
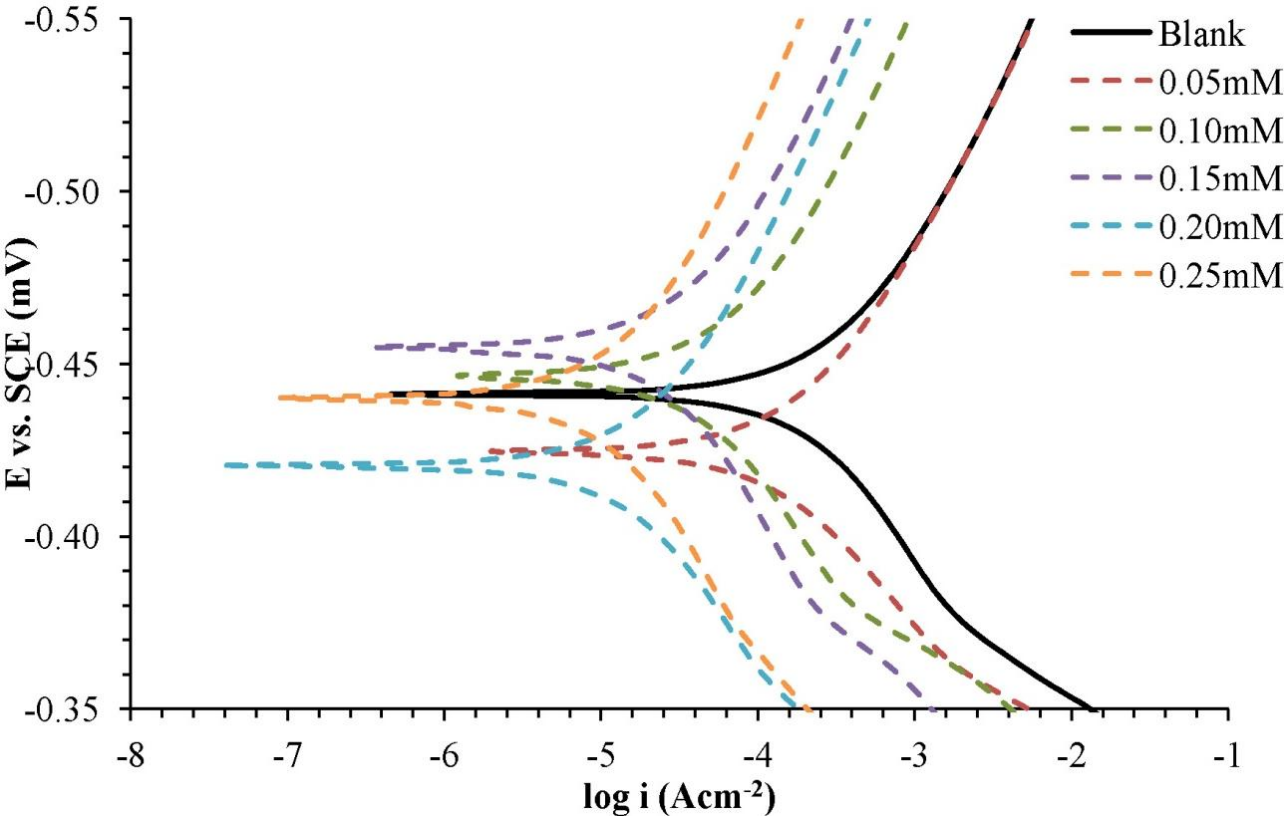
Potentiodynamic polarization curve for mild steel in 1.0 M H_2_SO_4_ with different concentrations of the corrosion inhibitor at 30 °C. SCE, saturated calomel electrode.

Based on Figure 3, the transition of values of *E_corr_* to more negative values were detected in different corrosion inhibitor concentrations. In addition, the cathodic and anodic current density also decreased with the increasing concentrations of the corrosion inhibitor. This point can be explained by the adsorption of corrosion inhibitor molecules on the sample surface, forming a protective mild steel surface. Based on Table 3, it was found that the anodic Tafel slope (βa) and the cathodic Tafel slope (βc) had changed with the addition of the corrosion inhibitors. This shows that the corrosion inhibitor influences the anodic and cathodic reactions. As shown in Table 3, the corrosion current density (*i_corr_*) became lower and the inhibition efficiency (*IE*) increased with increasing concentrations of the corrosion inhibitor. The corrosion inhibitor is effective in protecting mild steel in a H_2_SO_4_ solution.

**Figure 4.**
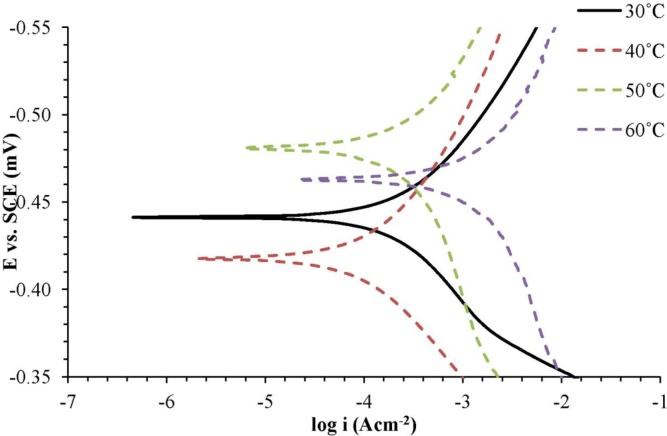
Potentiodynamic polarization curve for mild steel in 1.0 M H_2_SO_4_ with 0.25 mM of the corrosion inhibitor at different temperatures.

Based on Figure 4 and Table 4, it was found that the anodic Tafel slope (βa) and cathodic Tafel slope (βc) increased with the increasing of the temperature of the H_2_SO_4_ solution with 0.25 mM of the corrosion inhibitor. Corrosion current density (*i_corr_*) was higher, and the inhibition efficiency (*IE*) also decreased. Increasing temperatures of the H_2_SO_4_ solution with the corrosion inhibitors has resulted in corrosion inhibitor molecule desorption from the surface of the metal and caused corrosion of the metal occurring at a faster rate.

### 2.3. Adsorption Isotherm

**Figure 5.**
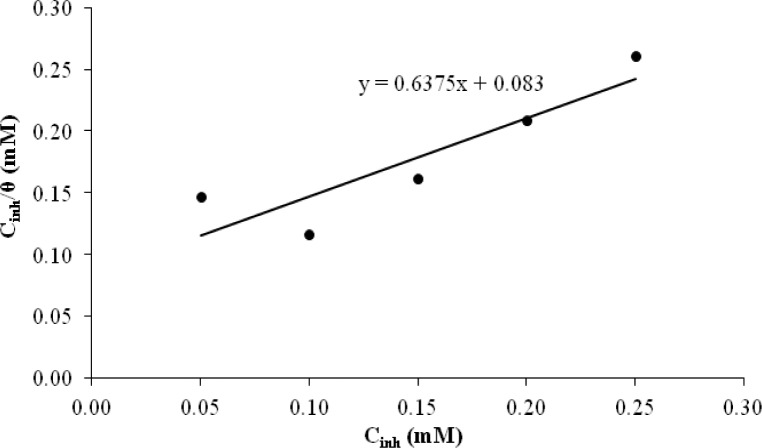
Adsorption isotherm for mild steel in 1.0 M H_2_SO_4_ with different concentrations of the corrosion inhibitor.

Adsorption isotherms can provide basic information about the interaction between the inhibitor and mild steel surface [21]. The corrosion inhibition of organic inhibitors on mild steel in sulfuric acid can be explained via the molecular adsorption method. The adsorption process is influenced by the structure of the organic compounds, the charge distribution in the molecules, the nature of the surface-charged metals and the types of media used [22]. The phenomenon of interaction between the metal surface and the inhibitor can be better understood in terms of the adsorption isotherm. The plots of $\frac{c_{inh}}{\theta }$ (Figure 5) against *C_inh_* yield a straight line with an approximately unit-slope, indicating that the inhibitor under study obeys the Langmuir adsorption isotherm [23], as in the equation below.

$$\frac{C_{inh}}{\theta }=\frac{1}{K_{ads}}+C_{inh}$$ (2)

where *C_inh_* is the concentration of the inhibitor and *K_ads_* is the adsorption constant obtained from the intercept of the straight line. *K_ads_* is associated with the standard free energy of adsorption, (Δ*G*^0^*_ads_*), where Δ*G*^0^*_ads_* is given as below:

$$\Delta G_{ads}^{0}=-\text{RT}\;\ln(55.5K_{ads})$$ (3)

where the value of 55.5 represents the molar concentration of water in solution expressed in units of M, R is the universal gas constant and *T* is the absolute temperature [24,25].

From Figure 5, the value of *k_ads_* and Δ*G*^0^*_ads_* was calculated. The value of *k_ads_* is 12,048.19 mol^−1^ dm^3^, while the value of Δ*G*^0^*_ads_* is −33.81 kJ mol^−1^. The negative value of Δ*G*^0^*_ads_* indicates the spontaneous adsorption of the corrosion inhibitor on the mild steel surface and the strong interaction between the inhibitor molecules and the surface of the mild steel corrosion. Generally, a value of Δ*G*^0^*_ads_* around −20 kJ/mol is consistent with physical adsorption, while a value of Δ*G*^0^*_ads_* around −40 kJ/mol or higher is chemical adsorption occurring with the sharing or transfer of electrons from organic molecules to the surface of the mild steel. The calculated value of Δ*G*^0^*_ads_* is around −40 kJ/mol and explains the adsorption mechanism of the corrosion inhibitor through chemical adsorption [26].

#### Corrosion Kinetic Parameters

The activation energy (*E_a_*) in the corrosion process is calculated based on the results of experimental measurements of potentiodynamic polarization. The correlation between the corrosion current density on corrosion temperature can be expressed by the Arrhenius equation. The Arrhenius equation is represented by the following equation [26]:

$$i_{corr}=A\text{exp}\;(\frac{-E_{a}}{RT})$$ (4)

where *i_corr_* is the corrosion current density in the A·cm^−2^, A is the electrochemical constant, *E_a_* is the activation energy in J·mol^−1^, *R* is the gas constant worth 8314 J·mol^−1^·K^−1^ and *T* is the temperature in units of K. The Arrhenius equation can be converted into logarithmic form and become the following equation:

$$\ln i_{corr}=(\frac{-E_{a}}{R})(\frac{1}{T})+\ln A$$ (5)

**Figure 6.**
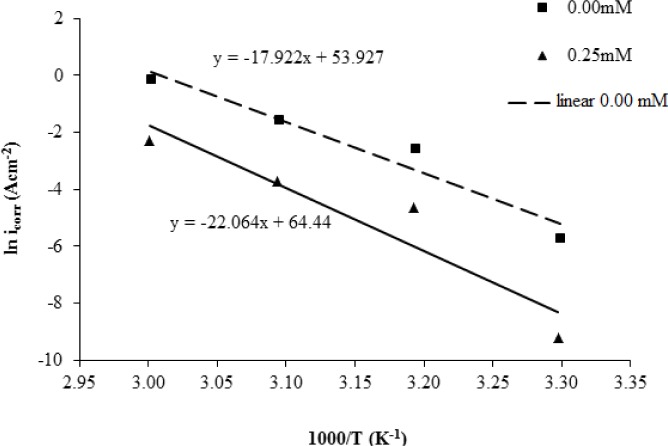
The Arrhenius plot for mild steel in 1.0 M H_2_SO_4_ with 0.00 mM and 0.25 mM concentrations of the corrosion inhibitor.

The Arrhenius plot represented by the graph of ln*i_corr_* against 1000/*T* is plotted based on the results of potentiodynamic polarization measurement. As shown in Figure 6, *E**_a_* can be calculated by using the slope of the graph and is shown in Table 5.

The following equation shows the Arrhenius equation transition state. The activation enthalpy and entropy of activation, Δ*H_a_* and Δ*S_a_*, are calculated by the Arrhenius equation [4]:

$$i_{corr}=\frac{RT}{Nh}\text{exp}(\frac{\Delta S_{a}}{R})\text{exp}(\frac{-\Delta H_{a}}{RT})$$ (6)

where *N* is Avogadro’s number valued 6.02 × 10^23^ mol^−1^, and *h* is the Plank constant, 6.63 × 10^−34^ m^2^·kg·s^−1^. The Arrhenius equation of state will transition into the following equation by using the algorithm:

$$\ln(\frac{i_{corr}}{T})=(\frac{-\Delta H_{a}}{R})(\frac{1}{T})+[\ln(\frac{R}{Nh})+\frac{\Delta S_{a}}{R}]$$ (7)

**Figure 7.**
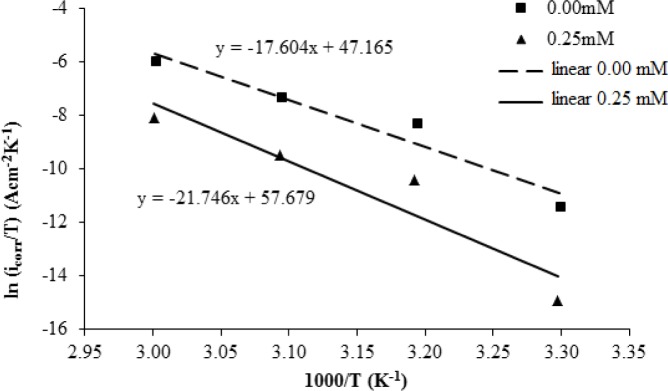
The situation plot for mild steel in 1.0 M H_2_SO_4_ with 0.00 mM and 0.25 mM concentrations of the corrosion inhibitor.

The plots of ln(*i_corr_*/*T*) *versus* 1000/*T* are shown in Figure 7. The graph is a straight line graph. The value of the enthalpy of activation, Δ*H_a_*, is calculated from the slope of the graph (−Δ*H_a_*/*R*), while the entropy of activation, Δ*S_a_*, is calculated from the intersection with the *y*-axis [ln(*R*/*Nh*) + (Δ*S_a_*/*R*)]. The values are shown in Table 5.

As shown in Table 5, the value of *E_a_* for mild steel in 1.0 M H_2_SO_4_ without any corrosion inhibitor is 149.00 kJ·mol^−1^, while for mild steel in 1.0 M H_2_SO_4_ with 0.25 mM of the corrosion inhibitor it is 183.44 kJ·mol^−1^. The increase in the value of the activation energy, *E_a_*, in the presence of corrosion inhibitors suggested the adsorption of the corrosion inhibitor on the surface of mild steel as 0.25 mM of the corrosion inhibitor is added in 1.0 M H_2_SO_4_. When the temperature increases, a reduction of the inhibitor adsorption on metal surfaces occurs. At higher temperatures, corrosion inhibitor molecule desorption occurs and causes mild steel surfaces to be exposed to corrosion. The values of *E_a_* also suggest that the inhibition process is a controlled surface reaction, because the values of *E_a_* for both situations, with and without the presence of a corrosion inhibitor, exceed 20 kJ·mol^−1^ [27].

The value of the enthalpy of activation, Δ*H_a_*, for mild steel in 0.25 mM of the corrosion inhibitor is higher than that without the corrosion inhibitor. This can be explained by the presence of the energy barrier for the reaction, which is the corrosion inhibitor adsorption process that led to the higher value of Δ*Ha* [28].

The value of the entropy of activation, Δ*S_a_*, increased when 0.25 mM of the corrosion inhibitor was added in 1.0 M H_2_SO_4_. The mild steel surface is covered by corrosion inhibitor molecules. This will slow down the release of hydrogen ions on the metal surface, causing the system to move from a more organized into a more random order and, thus, increasing the entropy of activation [29].

### 2.4. Electrochemical Frequency Modulation (EFM)

The experimental results of electrochemical frequency modulation (EFM) are the spectrum of current response as a function of frequency. This spectrum is known as the intermodulation spectrum. The spectrum containing the current response is used to determine the peak harmony current and peak intermodulation current. A larger peak was used to calculate the current density (*i_corr_*), Tafel slope (β_1_ and β_2_) and causality factor (*CF*-2 and *CF*-3). The electrochemical parameters were determined by Gamry Echem Analyst software [28]. Table 6 shows the EFM electrochemical parameters for mild steel in 1.0 M H_2_SO_4_ at 30 °C with different concentrations of the corrosion inhibitor, while Table 7 shows the EFM electrochemical parameters for mild steel in 1.0 M H_2_SO_4_ with 0.25 mM of the corrosion inhibitor at different temperatures.

Based on Table 6, the corrosion current density, *i_corr_*, decreased by increasing the concentration of the corrosion inhibitor. The inhibition efficiency (*IE*) is calculated by the following formula [29]:

$$IE_{EFM}(\%)=\frac{i_{corr}^{0}-i_{corr}}{i_{corr}^{0}}\times 100$$ (8)

where *i_corr_*^0^ and *i_corr_* are the corrosion current density values without and with the corrosion inhibitor, respectively.

The parameters in Tables 6 and 7 show that the data collected does not have a good quality if compared to the standard value of 2.0 for *CF-2* and 3.0 for *CF-3* [30]. If the *CF*s differ significantly from the theoretical value, it can be concluded that the measurements are affected by noise. When the difference in *CF*-2 and *CF*-3 is in the range of 0.2 and 0.3, the EFM data is accurate. The difference in the theoretical value of the *CF* may be caused by an amplitude perturbation that is too small or a spectral frequency resolution that is not high enough [26]. Values of *i_corr_* were converted to a corrosion rate with units of milli-inch per year (mpy). The corrosion rate as shown in Table 6 is low, with the increasing of the corrosion inhibitor concentration. The efficiency of the inhibition increased when the concentration of the corrosion inhibitor changed from 0.00 mM to 0.25 mM.

Based on Table 7, the data also had a poor quality when compared with the standard value of 2.0 for *CF*-2 and 3.0 for *CF*-3. The value of *i_corr_* increased with the increasing temperature of the H_2_SO_4_ solutions with 0.25 mM of the corrosion inhibitors. The corrosion rate became higher with increasing solution temperature. The inhibition efficiency became lower when the solution temperature increased from 30 °C to 60 °C.

### 2.5. The Mechanism of Inhibition

**Scheme 2.**
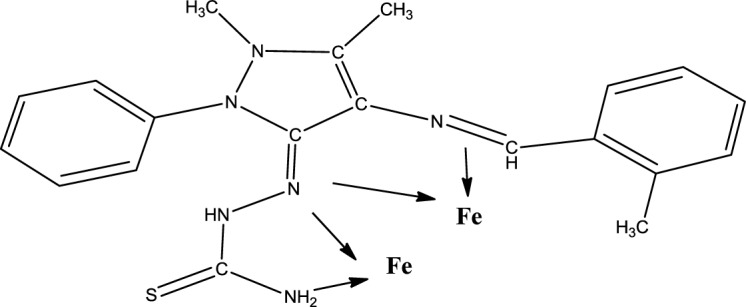
The mechanism of inhibition.

Generally, organic inhibitors are adsorbed on the metal surface and prevent further dissolution of metal by blocking either the cathodic or anodic reaction or both. Organic inhibitors, capable of forming insoluble complexes, or chelates, with metallic ions present on the surface of metal [31]. The inhibition efficiency of our corrosion inhibitor against the corrosion of steel in 1.0 M H_2_SO_4_ can be explained on the basis of the number of adsorption sites, their charge density, molecular size, mode of interaction with the metal surface and the ability to form a metallic complex. The π electrons and free electrons on the S and N atoms form bonds with the metal surface; see Scheme 2.

### 2.6. Scanning Electron Microscope

**Figure 8.**
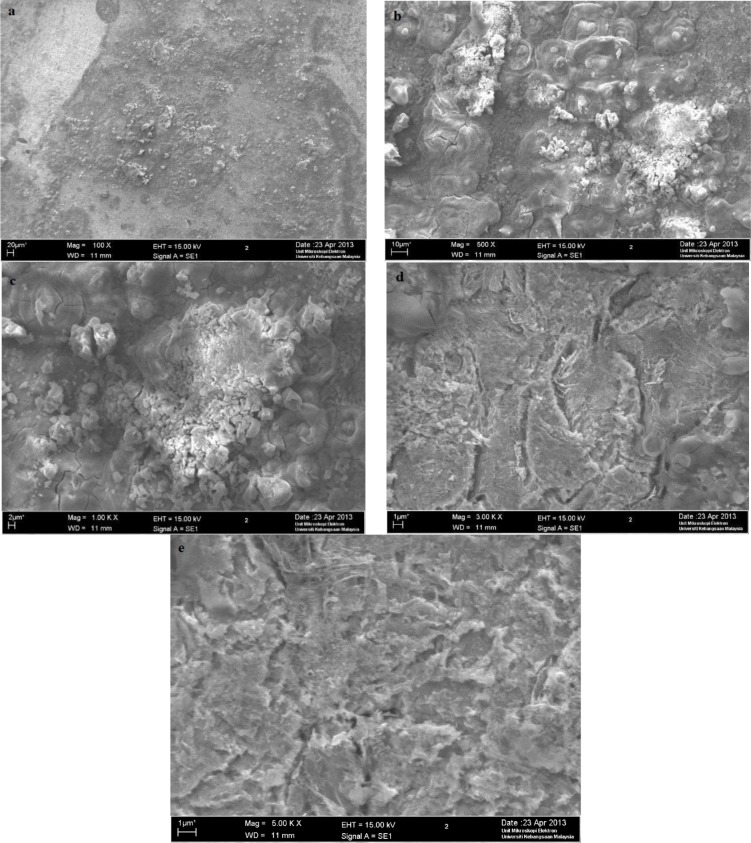
The SEM micrographs for mild steel in 1.0 M H_2_SO_4_ without the corrosion inhibitor at 30 °C. (**a**) 100×; (**b**) 500×; (**c**) 1000×; (**d**) 3000×; (**e**) 5000×.

**Figure 9.**
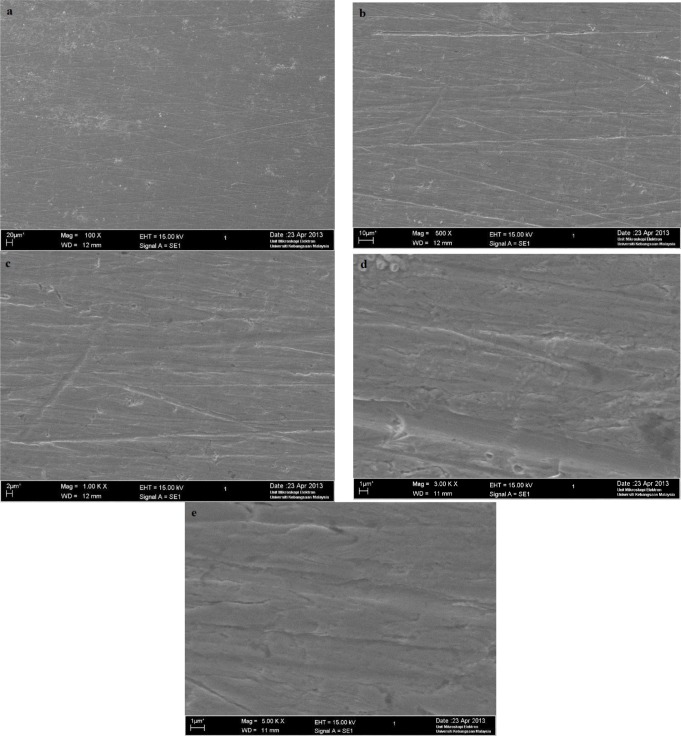
The SEM micrographs for mild steel in 1.0 M H_2_SO_4_ with 0.25 mM of the corrosion inhibitor at 30 °C. (**a**) 100×; (**b**) 500×; (**c**) 1000×; (**d**) 3000×; (**e**) 5000×.

A scanning electron microscope test was conducted at the UKM Electron Microscopy Unit. Based on Figure 8, as expected, serious corrosion of mild steel occurred where the mild steel surface, which was originally clean and smooth, became rough. The mild steel surface was significantly attacked by H_2_SO_4_. Based on Figure 9, the mild steel surface did not suffer serious corrosion. The corrosion inhibitor provided protection to the mild steel from the corrosion attack caused by H_2_SO_4_.

## 3. Experimental Section

All of the chemicals used in the present study were reagent grade (supplied Sigma-Aldrich, Kuala Lumpur, Malaysia) and were used as supplied without further purification. The FTIR spectra were measured using a Thermo Scientific Model Nicolate 6700 Spectrophotometer. NMR spectra were recorded on a model AVANCE III 600 MHz spectrometer.

### 3.1. Synthesis of Corrosion Inhibitor

The corrosion inhibitor was synthesized according to [32], and the structure was confirmed with elemental analyses and spectral analyses (IR, ^1^H-NMR).

### 3.2. Corrosion

Mild steel specimens obtained from the Metal Samples Company were used as the working electrodes throughout this study. The composition (wt%) of the mild steel was as follows: Fe, 99.21; C, 0.21; Si, 0.38; P, 0.09; S, 0.05; Mn, 0.05; and Al, 0.01; and it had an active surface area of 4.5 cm^2^. The specimens were cleaned according to the ASTM standard procedure, G1-03 [33]. The measurements were conducted in aerated, non-stirred 1.0 M sulfuric acid solutions at 30, 40, 50 and 60 °C at the following 2-(1,5-dimethyl-4-(2-methylbenzylidene)amino)-2-phenyl-1H-pyrazol-3(2H)-ylidene)hydrazinecarbothioamide corrosion inhibitor concentrations: 0 mM, 0.05 mM, 0.10 mM, 0.15 mM, 0.2 mM and 0.25 mM. The acid concentrations were selected based on the conditions commonly encountered during the pickling process in industrial facilities. The solutions were freshly prepared using distilled water. Each measurement was repeated three times, and only the average values were reported to verify the reproducibility of the experiments. The cell contained three electrodes, working, counter and reference, which consisted of mild steel, a graphite bar and a saturated calomel electrode (SCE), respectively. The potentiodynamic current-potential curves were swept from −0.2 to 0.2 V_SCE_ at a scan rate of 0.5 mV·s^−1^. The measurements were performed using the Gamry Instrument Potentiostat/Galvanostat/ZRA (REF 600) model. The DC105 and EIS300 software packages developed by Gamry were used to perform the potentiodynamic scan and the electrochemical impedance spectroscopy (EIS) measurements, respectively. The collection of the electrochemical measurements began approximately 30 min after the working electrode was immersed in the solution to allow for the stabilization of the steady-state potential.

### 3.3. Metallographic Examination

Scanning electron microscopy (SEM, TM1000 Hitachi Tabletop Microscope at 2000× magnification, was used to examine the mild steel samples that had been immersed in H_2_SO_4_, both with and without the corrosion inhibitor, for 3 h.

## 4. Conclusions

In this study, a 4-aminoantipyrine derivative, was synthesized and characterized using various spectroscopic methods. Changes in the electrochemical impedance spectroscopy (EIS), and potentiodynamic polarization were used to study the corrosion inhibition of mild steel in 1.0 M H_2_SO_4_ solutions at 30, 40, 50 and 60 °C, using 2-(1,5-dimethyl-4-(2-methylbenzylidene)amino)-2-phenyl-1H-pyrazol-3(2H)-ylidene)hydrazinecarbothioamide as an inhibitor. This compound exhibited excellent inhibition performance as a mixed-type inhibitor. In general, the acidic corrosion of mild steel was reduced by the addition of an appropriate concentration. The inhibition efficiencies increased with inhibitor concentration, but were reduced proportionally with temperature. The inhibition efficiencies obtained from the EIS data were comparable with those obtained from the polarization measurements in which the inhibitory solution had higher values than those of the non-inhibitory solution. The inhibitor act as an efficient corrosion inhibitor on a mild steel surface obeys the Langmuir adsorption isotherm. The SEM micrographs demonstrated that the inhibitor molecules form a protective film on the steel surface.0 M sulfuric acid and exhibits a maximum inhibition efficiency of 93%.

## Figures and Tables

**Table 1.** Data of constant phase element (*CPE*) matching for mild steel in 1.0 M H_2_SO_4_ with different concentration of the corrosion inhibitor at 30 °C. *R_s_*, solution resistance; *R_ct_*, charge transfer of resistance; *C_dl_*, double-layer charge; *IE*, inhibition efficiency (*IE*).

Concentration (mM)	*R_s_* (ohm cm^2^)	*R_ct_* (ohm cm^2^)	*CPE_dl_*		*C_dl_* (μF cm^−2^)	*IE* (%)
			Y_o_ (μS s^α^ cm^−2^)	α		
Blank	1.31	64.08	425.33	0.91	293.46	0.00
0.05	1.42	85.14	980.89	0.90	754.01	24.74
0.10	1.59	247.01	432.67	0.78	232.05	74.06
0.15	1.89	441.59	329.56	0.77	182.66	85.49
0.20	1.53	606.15	504.00	0.80	376.16	89.43
0.25	1.71	893.70	348.44	0.76	243.20	92.83

**Table 2.** Data for the *CPE* matching for mild steel in 1.0 M H_2_SO_4_ with 0.25 mM of the corrosion inhibitor at different temperatures.

Temperature (°C)	Concentration (mM)	*R_s_* (ohm cm^2^)	*R_ct_* (ohm cm^2^)	*CPE_dl_*		*C_dl_* (μF cm^−2^)	*IE* (%)
				Y_o_ (μS s^α^ cm^−2^)	α		
30	Blank	1.31	64.08	425.33	0.91	293.46	0.00
	0.25	1.71	893.70	348.44	0.76	243.20	92.83
40	Blank	1.21	9.34	4368.89	0.70	1135.42	0.00
	0.25	1.05	114.41	490.67	0.69	136.68	91.84
50	Blank	1.19	6.06	3882.22	0.70	774.62	0.00
	0.25	0.97	90.99	466.00	0.69	109.38	93.34
60	Blank	1.07	4.04	2962.22	0.72	539.09	0.00
	0.25	0.93	15.53	553.11	0.74	100.06	72.70

**Table 3.** Polarization parameters for mild steel in 1.0 M H_2_SO_4_ with different concentrations of the corrosion inhibitor at 30 °C. mpy, milli-inch per year.

Concentration (mM)	Potentiodynamic Polarization Measurement					
	β_a_ (V dec^−1^)	β_c_ (V dec^−1^)	*i_corr_* (μA cm^−2^)	−*E_corr_* (mV *vs*. SCE)	Corrosion Rate (mpy)	*IE* (%)
Blank	0.077	0.099	342.22	441.00	156.90	0.00
0.05	0.068	0.096	224.44	424.00	102.70	34.42
0.10	0.058	0.090	46.44	446.00	21.24	86.43
0.15	0.060	0.091	22.44	455.00	10.32	93.44
0.20	0.055	0.088	13.80	421.00	6.32	95.97
0.25	0.070	0.098	12.18	440.00	5.58	96.44

**Table 4.** Polarization parameters for mild steel in 1.0 M H_2_SO_4_ with 0.25 mM of the corrosion inhibitor at different temperatures.

Temperature (°C)	Concentration (mM)	Potentiodynamic polarization (PD) Measurement					
		β_a_ (mV dec^−1^)	β_c_ (mV dec^−1^)	*i_corr_* (mA cm^−2^)	−*E_corr_* (mV *vs*. SCE)	Corrosion Rate (mpy)	*IE* (%)
30	Blank	76.70	99.00	0.34	441.00	156.90	0.00
	0.25	70.20	98.10	0.01	440.00	5.58	96.44
40	Blank	99.70	138.30	7.94	425.00	807.80	0.00
	0.25	96.40	131.70	0.95	418.00	97.12	87.98
50	Blank	117.40	166.10	21.30	414.00	2,169.00	0.00
	0.25	202.60	150.20	2.40	481.00	244.40	88.73
60	Blank	189.70	286.30	87.70	415.00	8,925.00	0.00
	0.25	142.40	163.10	9.99	463.00	1,017.00	88.60

**Table 5.** Corrosion kinetic parameters for mild steel in 1.0 M H_2_SO_4_ without and with 0.25 mM of the corrosion inhibitor. *E_a_*, activation energy; Δ*H_a_*, activation enthalpy; Δ*S_a_*, entropy of activation.

Concentration	*E_a_* (kJ mol^−1^)	Δ*H_a_* (kJ mol^−1^)	Δ*S_a_* (J mol^−1^ K^−1^)
without inhibitor	149.00	146.36	194.59
0.25 mM of inhibitor	183.44	180.80	282.01

**Table 6.** Electrochemical frequency modulation (EFM) electrochemical parameters for mild steel in 1.0 M H_2_SO_4_ with different concentrations of the corrosion inhibitor at 30 °C. *i_corr_*, current density; β*_N_*, Tafel slope; *CF*, causality factor.

Concentration (mM)	*i_corr_* (μA cm^−2^)	β_1_ (mV dec^−1^)	β_2_ (mV dec^−1^)	Corrosion Rate (mpy)	*IE_EFM_* (%)	*CF*-2	*CF*-3
Blank	286.67	75.69	94.14	131.30	0.00	1.85	4.30
0.05	224.00	73.30	96.07	102.60	21.86	1.90	4.53
0.10	91.51	83.47	133.40	41.92	68.07	1.85	2.68
0.15	60.87	89.83	160.00	27.88	78.77	1.96	3.60
0.20	44.80	97.93	149.60	20.52	84.37	2.00	4.11
0.25	33.13	98.35	155.40	15.18	88.44	1.96	5.41

**Table 7.** EFM electrochemical parameters for mild steel in 1.0 M H_2_SO_4_ with 0.25 mM of the corrosion inhibitor at different temperatures.

Temperature (°C)	Concentration (mM)	*i_corr_* (mA cm^−2^)	β_1_ (mV dec^−1^)	β_2_ (mV dec^−1^)	Corrosion Rate (mpy)	*IE_EFM_* (%)	*CF*-2	*CF*-3
30	Blank	0.29	75.69	94.14	131.30	0.00	1.85	4.30
–	0.25	0.03	98.35	155.40	15.18	88.44	1.96	5.41
40	Blank	6.81	89.93	111.90	692.80	0.00	1.99	3.61
–	0.25	1.16	107.80	193.70	117.90	82.98	1.95	4.18
50	Blank	19.14	93.68	109.60	1,948.00	0.00	2.06	5.77
–	0.25	2.26	129.40	242.70	230.50	88.17	1.96	5.77
60	Blank	56.74	119.60	146.50	5,776.00	0.00	1.81	1.42
–	0.25	10.85	144.00	192.50	1,104.00	80.89	1.90	5.14
